# Cross-correlation analysis of blood and microdialysis-assessed tissue lactate monitoring: a study in critically ill septic patients

**DOI:** 10.1186/cc10867

**Published:** 2012-03-20

**Authors:** I Ilias, P Kopterides, N Nikitas, D Vassiliadi, M Theodorakopoulou, E Papadomichelakis, M Lygnos, A Flevari, M Rizos, F Frantzeskaki, C Diakaki, E Paramythiotou, E Dimitriadou, S Orfanos, A Armaganidis, I Dimopoulou

**Affiliations:** 1 'Elena' Hospital, Athens, Greece; 2 'Attiko' University Hospital, Haidari, Athens, Greece

## Introduction

In the critical care setting, blood lactate (BL) concentration is measured to assess - albeit indirectly - tissue oxygenation. In addition, serial BL measurements are clinically useful since a drop in BL is a good prognostic finding, whereas persistent BL elevation portends poor outcome. Microdialysis (MD) enables direct monitoring of tissue metabolic changes. This study aimed to describe the dynamics of MD-assessed tissue lactate (TL) *vis-à-vis *BL in septic patients with and without shock.

## Methods

We measured BL and thigh adipose tissue TL serially every 4 hours for 6 days in 88 patients with septic shock and 45 patients at various sepsis stages hospitalized in a tertiary-care hospital ICU. Analysis was done with measurement of the area under the curve (AUC) of lactate*hours, cross-approximate entropy (X-ApEn) and cross-correlation. Comparisons of septic shock versus nonseptic shock patients' results were done with *t *tests and *z *statistics.

## Results

BL and TL were higher in septic shock patients compared to nonseptic shock patients (AUCs of 276 vs. 176 and 355 vs. 273 mmol/l*hours, respectively; Welch's *t *test: *P *< 0.0001). X-ApEn for MDL/BL was lower in septic shock patients compared to those without septic shock (mean ± SD: 0.79 ± 0.12 vs. 1.14 ± 0.13, respectively; *t *test: *P *< 0.0001). Cross-correlation of TL versus BL was stronger in septic shock patients, with TL leading BL by 4 hours compared to TL versus BL with no lag time (*r *= +0.85, *P *< 0.0001 and *r *= +0.66, *P *< 0.0001, respectively) than in nonseptic shock patients (*r *= +0.58, *P *= 0.0003 with TL leading BL by 4 hours and *r *= +0.66, *P *< 0.0001 with no lag time; *z *statistic = 2.41 and *P *= 0.016 for leading BL compared to *z *statistic = 0.036, *P *= 0.971 for no lag time).

## Conclusion

In septic shock patients, tissue lactate levels - measured by MD - are higher compared to nonseptic shock patients. Furthermore, TL is better correlated with and precedes - within 4 hours - BL in septic shock patients compared to nonseptic shock patients. Further studies are warranted to assess the clinical value of serial TL monitoring.

